# Clinical Research Quo Vadis? Trends in Reporting of Clinical Trials and Observational Study Designs Over Two Decades

**DOI:** 10.14740/jocmr2115w

**Published:** 2015-04-08

**Authors:** Moritz C. Wyler von Ballmoos, James H. Ware, Bernhard Haring

**Affiliations:** a Department of Surgery & Division of Cardiothoracic Surgery, Medical College of Wisconsin, Milwaukee, 53232 WI, USA; b Department of Biostatistics, Harvard School of Public Health, Boston, 02115 MA, USA; c Comprehensive Heart Failure Center, Department of Internal Medicine I, University of Wurzburg, Wurzburg, Germany

**Keywords:** Clinical trials, Observational studies, Study design, Trend, Clinical research

## Abstract

**Background:**

Multiple classifications have been developed that classify the medical literature into different levels of evidence to facilitate the evaluation of study results and practice of evidence-based medicine. The suggested hierarchies of evidence are generally based on the type of study design; randomized, controlled clinical trials constitute the top level of evidence while case reports rank the lowest among epidemiologic study designs. However, little is known about the frequency with which different study designs appear in the medical literature overall. The purpose of this study was to describe trends in the frequency of reports of randomized control trials (RCTs) as compared to other study designs in the medical literature over two decades.

**Methods:**

Data about the prevalence of various types of study designs in the medical literature over the last two decades (years 1990 - 2009) were abstracted from PubMed, validated and subjected to cross-sectional and longitudinal analysis.

**Results:**

In the last 20 years, the annual rate of publication of journal articles has more than doubled. During this period, the percentage of observational studies increased from 29.9% to 40.5%, the percentage of reports of RCTs increased minimally, and there was a striking decline in the percentage of case reports (from 49.8% to 33.6%) in the medical literature overall. In contrast, in three selected, highly cited medical journals, the percentage of reports of RCTs increased by almost 10%. Surprisingly, the percentage of articles classified as case reports also increased (from 36.3% to 43.8%) in these three journals, while the percentage of reports of cohort and case-control studies decreased.

**Conclusion:**

Though the relative frequency of reports from RCTs has not changed substantially in the last 20 years, cohort studies and case-control studies have largely supplanted simple case reports. In contrast, in high impact journals, the representation of RCTs and case reports has increased, with corresponding declines in reports based on other study designs. Further research will be needed to determine whether those trends in publication have resulted in more robust evidence and faster advancement of medical knowledge.

## Introduction

Over the last several decades, clinical research and epidemiology have had a significant impact on clinical medicine, accounting for substantial advances in disease prevention and treatment. As part of this evolution, the field of epidemiology has given rise to a variety of study designs tailored towards special research needs and conditions like low incidence of cases or rare exposure. In order to provide patients with the best possible care, clinicians rely on high-quality research reports based on these different study designs to guide their medical decisions.

Multiple institutions have established classifications of evidence into hierarchical levels to help with the application of study results to evidence-based clinical practice. For such categorization, the strength of evidence of a given study is generally based upon its epidemiologic study design (Table 1). Although randomized clinical trials (RCTs) are now widely accepted as the gold standard for causal inference [1] and constitute the highest level of evidence in such classifications, specific circumstances in clinical medicine and research settings sometimes prevent the use of RCTs to investigate exposures as risk factors and explore benefits and risks of new treatment strategies [2-5]. Apart from cost issues, most commonly this is because randomization of the exposure is not feasible [6]. Major surgical interventions, long latent periods, rare events, and unethical withholding of treatment or purposeful exposures to a noxious agent are just a few examples of settings where observational study designs available in clinical epidemiology are better suited for investigating a suspected association or causal relationship [7]. Furthermore, clinical trials often require a more significant compromise between design for efficacy and generalizability of the results than other study designs [8].

**Table 1:** The Strength of Evidence

Level of evidence	Study design
1	RCTs
2	Cohort studies
3	Case-control studies
4	Case series/case reports
5	Narrative (literature) reviews, editorials

This table outlines the traditional hierarchy of study designs and evidence levels. The table is simplified and contains major categories of study designs only. Adopted from the Oxford Centre for Evidence-based Medicine (http://www.cebm.net/).

Validity of comparison, which is fundamental to causal inference, is achieved under the fewest assumptions by a diligently planned and carefully conducted RCT. This may explain why RCTs have been popularized for directing clinical decision making, have been moved to the top of the evidence ladder by many consortia (Table 1), and have received much attention in the medical literature. At the same time, epidemiology, biostatistics, and related sciences have developed new methods to increase the validity and improve statistical analysis of data gathered from observational studies, thereby increasing their importance as strategies for generating evidence that is just as important as that from RCTs [9, 10].

Currently hierarchical classifications of evidence based on study design are widely used but little information is available about recent trends in the use of these different study designs in the medical literature. In 1979, Fletcher and Fletcher presented a 30-year perspective on designs of clinical studies published in three selected medical journals between 1946 and 1976 [11]. This review was updated by McDermott et al in 1995 for the years 1971 - 1991 [12] again based on an analysis of three leading medical journals. In addition, several other investigators conducted analysis of temporal trends in study designs for select years and medical specialties [13-15]. With the introduction of online databases like PubMed, tools have become available for conducting more comprehensive bibliographic research. In the present study, we investigate trends in the use of different study designs in clinical research over the last 20 years (1990 - 2009) by reviewing the published literature reporting on human subjects research. To test the hypothesis that the publication of RCTs is increasing in high-impact journals, we also reviewed publications in three leading journals (the New England Journal of Medicine (NEJM), the Lancet and the Journal of the American Medical Association (JAMA)) and compared trends in these journals to the trends in the general literature indexed in PubMed in the same time period.

## Methods

### Bibliographic review of study design prevalence

In consultation with a professional librarian and using previously published search-term methods [16], the frequency of study designs in the published literature was assessed using a PubMed query. When research reports on human subjects are indexed in PubMed, they are assigned medical subject headings including a classification into specific research designs. The categories for classification include among others: RCTs, non-RCTs, cohort studies, case-control studies, cross-sectional studies, case series or reports, editorials, letters, systematic reviews and meta-analysis. Furthermore, primary analyses, secondary analyses, and repetitious reports are distinguished as part of this process.

Indexers at the United States National Library of Medicine (NLM) categorize each publication using classifications published online (http://www.nlm.nih.gov) that include descriptors for study designs and assign the corresponding publication type term. Our search for specific study designs and abstraction of the absolute number of studies published each year and for each study design were based on this classification. In a first search, all journals indexed in PubMed for the years 1990 - 2009 were screened for publications that reported results from human RCTs, non-RCTs, cohort and case-control studies, cross-sectional studies or case reports without any language or other restriction. In a subsequent step, the search was restricted to the NEJM, The Lancet, and the JAMA.

The appropriateness of the PubMed classification of research reports by study design has not to our knowledge been previously studied. In order to measure accuracy and potential misclassification occurring with this method, we conducted a validation study with a subsample of the analyzed data. Using a random number generator, 21 journal issues and 125 studies published during the time period of interest were retrieved from the archives of NEJM, JAMA and the Lancet, hand-searched, and classified based on the study classification suggested by Rothman et al [17]. These results were then compared to the PubMed data.

### Statistical analysis

Data were analyzed using SAS statistical software, version 9.2 (SAS Institute Inc., Cary, NC), and are presented as proportions or counts and 95% confidence intervals unless stated otherwise. Exact confidence intervals are reported. Pearson’s correlation coefficient was calculated as a measure of association. Proportions were compared by Pearson’s Chi-square statistic with continuity correction and the Bonferroni correction was used to account for multiple testing where appropriate. Generalized estimating equations using the independence assumption were used to perform cross-sectional comparisons and characterize temporal trends (P-values for trend). All data were abstracted from the respective online resources in duplicate by two investigators individually (B.H., M.W.v.B.). Concordance between data obtained from PubMed and the hand-search of study designs was analyzed as proportion of misclassification with exact 95% confidence intervals and by Cohan’s kappa coefficient. Overall, there was a misclassification of 4% (1.3-9.0%) of studies with two non-RCTs, one cohort, one case-control and one cross-sectional study that were not captured or misclassified by PubMed. This corresponds to an agreement coefficient of κ of 0.94 (P < 0.001), sensitivity of 97.6% (93.1-99.5%) and specificity of 96.9% (89.2-99.6%) of the PubMed data compared to hand searching and classification of studies. Statistical significance was inferred at a two-sided value of P < 0.05.

## Results

### Representation of different study designs in the medical literature

Including all types of publications, more than 332,000 studies were published between January 1, 1990 and December 31, 2009. The number of publications per 5-year period increased from 58,163 between 1990 and 1994 to 118,109 publications between 2005 and 2009 (Table 2).

**Table 2:** Research Designs Published in PubMed-Indexed Journals Between 1990 and 2009

Study design	1990 - 1994	1995 - 1999	2000 - 2004	2005 - 2009	P-trend
	%/N (95% CI)	%/N (95% CI)	%/N (95% CI)	%/N (95% CI)	
Randomized clinical trials	9.5% (8.8 - 10.3) 5,679 (4,810 - 6,548)	10.1% (9.8 - 10.4) 7,099 (6,561 - 7,638)	10.1% (9.7 - 10.5) 8,984 (7,290 - 10,678)	10.3% (10.1 - 10.5) 12,140 (10,699 - 13,580)	0.052 (r = 0.57)
Clinical trials	8.5% (5.0 - 12.1) 5,020 (2,042 - 7,999)	11.8% (11.4 - 12.3) 8,312 (7,437 - 9,188)	10.7% (9.8 - 11.5) 9,217 (7,441 - 10,993)	9.6% (8.3 - 11.0) 11,323 (9,879 - 12,766)	0.99 (r = 0.06)
Cohort and case-control studies	29.9% (27.4 - 32.4) 17,330 (15,076 - 19,583)	24.5% (33.5 - 35.4) 24,193 (22,014 - 26,371)	37.8% (36.7 - 38.8) 32,526 (27,513 - 37,539)	40.5% (39.4 - 41.6) 47,871 (42,804 - 52,938)	< 0.001 (r = 0.97)
Cross-sectional studies	2.3% (1.9 - 2.6) 1,313 (982 - 1,644)	2.9% (2.6 - 3.1) 2,021 (1,707 - 2,335)	3.9% (3.5 - 4.4) 3,430 (2,536 - 4,324)	6.0% (5.3 - 6.8) 7,146 (5,548 - 8,745)	< 0.001 (r = 0.96)
Case reports	49.8% (47.0 - 52.6) 28,821 (27,745 - 29,897)	40.7% (39.4 - 41.9) 28,476 (27,670 - 29,281)	37.5% (35.6 - 39.5) 32,111 (30,452 - 33,770)	33.6% (32.9 - 34.2) 39,629 (36,652 - 42,605)	< 0.001 (r = -0.95)
Total	58,163	70,101	86,268	118,109	

Numbers are counts and averaged proportions per year for each time period. r is correlation coefficient for study design frequency and year of publication. P-value is for time trend between 1990 and 2009.

**Figure 1:**
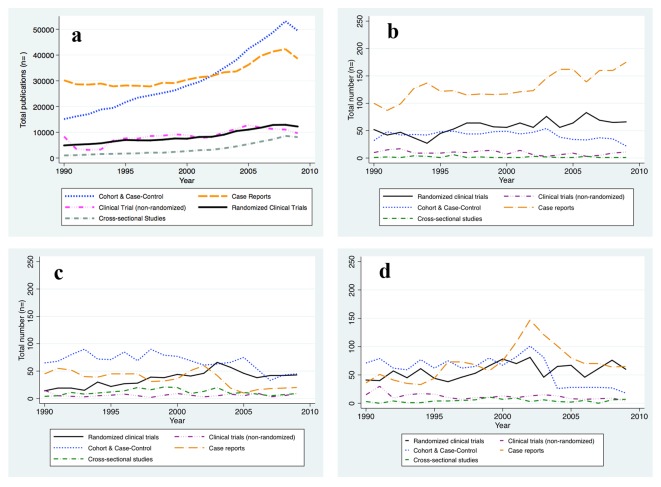
New reports on human subjects research by design and per year. (a) All Journals indexed in PubMed. (b) The New England Journal of Medicine. (c) The Journal of the American Medical Association. (d) The Lancet. The absolute number of human subjects research reports published per year is graphed for the time period between 1990 and 2009 and per study design, respectively. The number of reports publised increased for all study designs considered.

There were clear trends over that period in the relative frequency of reports of different types of study designs (Fig. 1 and Table 2). Cohort and case-control studies made up 29.9% of all reports in the early nineties (1990 - 1994) and increased to 40.5% of reports in the years 2004 - 2009 (P-trend < 0.001). A less dramatic increase was seen for RCTs (from 9.5% to 10.3%; P-trend: 0.052) and for the cross-sectional study design (from 2.3% to 6.0%; P-trend < 0.001), while the percentage of non-RCTs remained relatively constant (P-trend: 0.99) during the 20-year period. The increase in reports of cohort and case-control studies as well as RCTs was offset by a substantial reduction in the percentage of case reports in the literature between 1990 and 2009 (Table 2).

We hypothesized that the movement towards evidence-based medicine that has occurred in recent years would be reflected in an increase in reports of data obtained from study designs that rank higher in the hierarchy of evidence (Table 1). To this end we conducted the same analysis restricted to articles that had appeared in three respected and widely circulated medical journals (The Lancet, NEJM, and JAMA). In contrast to the findings obtained from the unrestricted PubMed search, we found a striking increase in frequency of reports from RCTs in these journals (from 20.3% to 30.9%; P-trend < 0.001) between 1990 and 2009. During the same period, reports of clinical trials without randomization decreased (from 6.6% to 3.7%; P-trend = 0.001). Surprisingly, there was also a notable increase in the number of case reports (from 36.3% to 43.8%; P-trend < 0.001), while reports of cohort and case-control studies became less common (from 34.6% to 19.2%; P-trend < 0.001) (Table 3).

**Table 3:** Research Designs Published in JAMA, NEJM and The Lancet Between 1990 and 2009

Study design	1990 - 1994	1995 - 1999	2000 - 2004	2005 - 2009	P-trend
	%/N (95% CI)	%/N (95% CI)	%/N (95% CI)	%/N (95% CI)	
Randomized clinical trials	20.3% (18.5 - 22.0) 109 (95 - 123)	22.5% (19.8 - 25.3) 137 (109 - 164)	25.9% (24.3 - 27.5) 180 (174 - 187)	30.9% (29.8 - 31.9) 174 (166 - 182)	< 0.001 (r = 0.91)
Clinical trials	6.6% (4.9 - 8.3) 36 (24 - 47)	4.5% (3.8 - 5.2) 27 (23 - 32)	3.8% (3.1 - 4.5) 26 (22 - 31)	3.7% (3.2 - 4.2) 21 (17 - 25)	0.001 (r = -0.74)
Cohort and case-control studies	34.6% (33.4 - 35.8) 186 (173 - 199)	32.1% (30.6 - 33.6) 194 (175 - 213)	26.4% (23.2 - 29.5) 185 (146 - 224)	19.2% (16.5 - 21.9) 108 (84 - 132)	< 0.001 (r = -0.94)
Cross-sectional studies	2.1% (1.5 - 2.8) 12 (7 - 16)	4.1% (3.4 - 4.8) 25 (18 - 32)	3.1% (2.2 - 3.9) 21 (13 - 29)	2.4% (1.7 - 3.1) 13 (8 - 19)	0.99 (r = 0.01)
Case reports	36.3% (35.2 - 37.5) 195 (182 - 209)	36.7% (33.8 - 39.5) 221 (202 - 240)	40.8% (37.2 - 44.4) 286 (238 - 333)	43.8% (41.5 - 46.1) 246 (229 - 262)	< 0.001 (r = 0.77)
Total	538	604	698	562	

Numbers are counts and averaged proportions per year for each time period. r is correlation coefficient for study design frequency and year of publication. P-value is for time trend between 1990 and 2009.

## Discussion

Previous studies of the frequency of research designs published in JAMA, NEJM and Lancet showed that between 1946 and 1976, study designs such as cross-sectional studies and case reports appeared more frequently in the medical literature than RCTs and cohort studies, a trend that the authors then claimed to deserve critical attention [12]. Here we provide an update and extension of this research conducted more than 30 years ago using the entire body of literature index in PubMed, which is a larger and probably more representative sample of the medical literature than has previously been used for such analyses.

This review of the published medical literature shows that publications reporting on human subjects research work in the general medical literature increasingly used population-based studies such as cohort or case-control studies while trending away from simple case reports, which usually allow for hypothesis generation at best and are typically considered the lowest level of evidence. RCTs that are situated at the other end of the hierarchical evidence ladder have also become more common in the literature between 1990 and 2009, although to a much lesser extent than cohort or case-control studies. The literature of today, compared to 20 years ago when many reports were simple case reports or case series, suggests a trend towards the use of study designs that rank higher in currently widely used classifications of evidence into hierarchical levels. Assuming that the most widely circulated and cited journals are a particularly efficient means of communicating novel approaches to problems in clinical practice, we hypothesized that these journals would reflect the trend away from low-grade evidence towards large trials and observational studies prominently. Indeed, the proportion of publications on RCTs in the NEJM, JAMA and The Lancet has exceeded the average in the medical literature by far and at all times during the analyzed time period. Furthermore, non-RCTs have become less frequent and these journals started publishing a much higher proportion of case reports but fewer cohort and case-control studies than other journals between 1990 and 2009.

These findings are intriguing. Most importantly our data suggest that, in contrast to the trend in the general body of medical literature, case-control and cohort studies rather than case reports have been pushed aside in high impact journals in order to make room for RCTs.

The recent interest in RCTs is not surprising. Most statistical and epidemiologic complexities of an RCT are implicit in the design, conduct and monitoring of the study. But once the data of an RCT are collected, analysis is usually straightforward. Importantly, solely RCTs can resolve the problem of confounding entirely and without further assumptions about the distribution of common causes (i.e. confounders). Establishing valid associations and cause-effect relationships is a more complex and delicate task with data from observational studies. Yet, no one will doubt the importance and impact that studies such as the Framingham Heart Study or Nurses’ Health Study, both cohort studies and not RCTs, have had on clinical medicine to date.

The paucity of reports from case-control and cohort studies in the highest impact medical journals may have several explanations. For one, there has always been a ferocious debate about the value of RCTs and observational studies based on different beliefs among authorities in the fields of epidemiology and clinical investigation. This debate has been furthered stimulated by publication of conflicting information from observational studies and RCTs on the same topic. One prominent example is the reports on risks and benefit of post-menopausal hormone replacement therapy in women. While initial data from a large and reputable cohort study suggested that HRT is beneficial [18, 19], the Women’s Health Initiative investigators subsequently reported that HRT cannot be unequivocally recommended in postmenopausal women and actually carries an increased risk of certain cardiovascular events and cancers [20, 21]. Critics of observational study designs took this as evidence against the validity of cohort studies in general. However, further evaluation of this apparent contradiction suggested that the two approaches and study designs provided answers to different questions, rather than actually being contradictory [22, 23]. Another reason for the low representation of observational studies in high impact journals may be the challenges of implementation of sound methods of observational study design and analysis that allow reliable inferences about causal relationships. This hypothesis raises the question whether a lack of expertise and funding mechanisms in the arena of observational study designs currently exists.

The present review of the medical literature also has limitations that must be given consideration when interpreting the results. First, the results are based on data from PubMed, which is one of the most comprehensive medical literature databases but does not include all available medical journals. This database was chosen due to the capability to identify multiple research designs in a highly reliable and reproducible fashion. In our validation study, we found excellent sensitivity and specificity of the PubMed search for the study designs under investigation. Though the chosen approach is vulnerable to some misclassification, this is likely to be non-differential. Another limitation is that our results are based on the classification of reports in terms of study design alone. We did not evaluate the study quality of individual reports. The latter is a critical issue when judging the strength of evidence a study provides. For example, a diligently planned, conducted and analyzed case-control study may provide better evidence than an RCT on the same topic that is affected by unsuccessful randomization, poor adherence, and substantial attrition. Furthermore, we did not assess the specific type of exposure and outcomes under investigation for individual studies which may possibly explain some of the differences found between the general literature and high impact journals. Such detailed analysis of studies was beyond our objective, but it is noteworthy that our approach falls short of accounting for this fact and thus does not provide the complete picture of the trend in low- versus high-grade evidence in the medical literature over the last decades. While interpreting the data one should also consider that some study designs are unsuitable for addressing certain questions entirely. For example, an RCT would never be the preferred study design for surveying prevalence of a disease. Lastly, publication bias, typically occurring secondary to non-publication of studies showing no difference between groups of comparison, may have affected the results reported in this study.

In summary, available data suggest that the rapidly growing body of research on human subjects has transitioned from reports considered lower-grade to reports of higher-grade evidence over time as based on the epidemiologic study design being used. However, the predominant research design differs in three selected highly cited journals as compared to PubMed listed journals in general. Whereas the trend in JAMA, NEJM and Lancet is in favor of RCTs and case reports, case-control and cohort studies are the prevailing study design in the medical literature on a larger scale.

## Abbreviations

CT: clinical trial
RCT: randomized clinical trial
